# Transposon Mutagenesis-Guided CRISPR/Cas9 Screening Strongly Implicates Dysregulation of Hippo/YAP Signaling in Malignant Peripheral Nerve Sheath Tumor Development

**DOI:** 10.3390/cancers13071584

**Published:** 2021-03-30

**Authors:** Germán L. Vélez-Reyes, Nicholas Koes, Ji Hae Ryu, Gabriel Kaufmann, Mariah Berner, Madison T. Weg, Natalie K. Wolf, Susan K. Rathe, Nancy Ratner, Branden S. Moriarity, David A. Largaespada

**Affiliations:** 1 Masonic Cancer Center, University of Minnesota, Minneapolis, MN 55455, USA; koesx006@umn.edu (N.K.); ryuxx083@umn.edu (J.H.R.); kaufm272@umn.edu (G.K.); berne068@umn.edu (M.B.); wegxx001@umn.edu (M.T.W.); wolfx449@umn.edu (N.K.W.); rath0096@umn.edu (S.K.R.); mori0164@umn.edu (B.S.M.); larga002@umn.edu (D.A.L.); 2 College of Medicine, University of Cincinnati, Cincinnati, OH 45267, USA; nancy.ratner@cchmc.org; 3 Division of Experimental Hematology and Cancer Biology, Cincinnati Children’s Hospital Medical Center, Cincinnati, OH 45267, USA; 4 Department of Pediatrics, University of Minnesota School of Medicine, Minneapolis, MN 55455, USA

**Keywords:** neurofibromatosis Type 1, cancer biology, genetic screen

## Abstract

**Simple Summary:**

Malignant peripheral nerve sheath tumors (MPNSTs) are highly aggressive tumors with a complex genetic landscape. Patients with neurofibromatosis type 1 syndrome (NF1) are at a high risk of MPNSTs, which usually develop from pre-existing benign Schwann cell tumors called plexiform neurofibromas. In this study, we aimed to find genes that, when altered, resulted in MPNST development. Our results suggest that the functional genetic landscape of human MPNST is complex and implicates the hippo/Yes Activated Protein (YAP) pathway in the transformation of neurofibromas.

**Abstract:**

Malignant peripheral nerve sheath tumors (MPNSTs) are highly aggressive, genomically complex, have soft tissue sarcomas, and are derived from the Schwann cell lineage. Patients with neurofibromatosis type 1 syndrome (NF1), an autosomal dominant tumor predisposition syndrome, are at a high risk for MPNSTs, which usually develop from pre-existing benign Schwann cell tumors called plexiform neurofibromas. NF1 is characterized by loss-of-function mutations in the *NF1* gene, which encode neurofibromin, a Ras GTPase activating protein (GAP) and negative regulator of RasGTP-dependent signaling. In addition to bi-allelic loss of *NF1*, other known tumor suppressor genes include *TP53*, *CDKN2A*, *SUZ12*, and *EED*, all of which are often inactivated in the process of MPNST growth. A sleeping beauty (SB) transposon-based genetic screen for high-grade Schwann cell tumors in mice, and comparative genomics, implicated Wnt/β-catenin, PI3K-AKT-mTOR, and other pathways in MPNST development and progression. We endeavored to more systematically test genes and pathways implicated by our *SB* screen in mice, i.e., in a human immortalized Schwann cell-based model and a human MPNST cell line, using CRISPR/Cas9 technology. We individually induced loss-of-function mutations in 103 tumor suppressor genes (TSG) and oncogene candidates. We assessed anchorage-independent growth, transwell migration, and for a subset of genes, tumor formation in vivo. When tested in a loss-of-function fashion, about 60% of all TSG candidates resulted in the transformation of immortalized human Schwann cells, whereas 30% of oncogene candidates resulted in growth arrest in a MPNST cell line. Individual loss-of-function mutations in the *TAOK1*, *GDI2*, *NF1*, and *APC* genes resulted in transformation of immortalized human Schwann cells and tumor formation in a xenograft model. Moreover, the loss of all four of these genes resulted in activation of Hippo/Yes Activated Protein (YAP) signaling. By combining *SB* transposon mutagenesis and CRISPR/Cas9 screening, we established a useful pipeline for the validation of MPNST pathways and genes. Our results suggest that the functional genetic landscape of human MPNST is complex and implicate the Hippo/YAP pathway in the transformation of neurofibromas. It is thus imperative to functionally validate individual cancer genes and pathways using human cell-based models, to determinate their role in different stages of MPNST development, growth, and/or metastasis.

## 1. Introduction

Plexiform neurofibromas are a common manifestation of neurofibromatosis type 1 syndrome (NF1). NF1 is caused by the inheritance of one mutant and non-functional copy of the *NF1* gene. *NF1* encodes neurofibromin, a Ras GTPase activating protein (GAP) and negative regulator of RasGTP-dependent signaling pathways. Roughly 50% of NF1 patients have a plexiform neurofibroma, which show a loss of the wild type *NF1* allele in a Schwann lineage cell^1^. Plexiform neurofibromas (PNF) can be present at birth and many malignant peripheral nerve sheath tumors (MPNSTs) form from pre-existing PNFs [1]. Plexiform neurofibromas are composed of a variety of cell types, including neurons, endothelial cells, fibroblasts, mast cells, macrophage, and Schwann cells, all of which are the neoplastic components of these tumors. Some of these cells are not part of the tumor per se, but act as tumor supporting cells. Although MPNSTs affect only about 0.001% of the general population, NF1 patients face dramatically increased risk, and MPNST is the most common cause of death in adults with NF1. It is estimated that about 10–15% of all patients with NF1 will develop an MPNST in their lifetime [2]. 

As in plexiform neurofibromas, many MPNSTs have biallelic inactivation of the *NF1* gene [3]. Ras hyperactivation, caused by loss of *NF1*, does not result in the malignant transformation of neurofibromas [4]. Transformation of the benign plexiform neurofibroma to an “atypical” neurofibroma typically includes *CDKN2A/CDKN2B* gene loss [4]. *CDKN2A/2B* loss together with *TP53*, *RB1*, and the polycomb repressor complex genes *EED* or *SUZ12* are hallmarks of MPNSTs. MPNST progression likely involves additional genetic changes including gene copy number alterations (CNAs) and epigenetic alterations. In fact, MPNSTs are classified as “Type C” tumors, dominated by recurrent gene copy number alterations (CNAs) rather than recurrent single nucleotide variants (SNVs) [5]. As described by the The Cancer Genome Atlas (TCGA) consortium and previous work, MPNSTs are characterized by a high number of recurrent chromosomal alterations causing CNAs affecting many genes, while harboring a minimal number of recurrent mutations and few defined examples of activated oncogenes [6]. Thus, the spectrum of changes that drive the genetic evolution to MPNST is difficult to define using human genomic data alone. Instead, functional data must be added. The definition of these driver alterations opens new avenues for therapy, which are desperately needed.

Currently, there are limited targeted therapies available to treat MPNSTs. Physicians rely on standard chemotherapy—often ifosfamide and doxorubicin—and radiation, with surgical resection, when possible [7,8]. Inhibitors of kinases activated downstream of Ras-GTP, such as PI3K, MEK, and mTOR, have been proposed from human and animal models, but no positive results have been reported in human trials [9,10]. To identify pathways, we performed a sleeping beauty (*SB*) transposon-based forward genetic screen for low- and high-grade Schwann cell tumors in mice [11]. In addition to the known Schwann cell tumor suppressor genes (TSGs), we identified *NF1*, *NF2*, *PTEN*, and the pathways that they are involved in. Many of these candidate genes were found to be altered recurrently at the gene expression, gene copy number, or methylation states in human MPNSTs. In this manuscript, we describe a secondary, human Schwann cell-based genetic screen, motivated by specific genes and pathways altered in this Schwann cell tumor screen in mice performed by our group using the *SB* transposon [11]. We validated the role of Hippo, Wnt/β-catenin, and Rho signaling, as well as other genes, in human Schwann cell tumors and discuss new approaches toward the treatment of MPNSTs.

## 2. Results

### 2.1. CRISPR/Cas9-Based Secondary Cancer Gene Screening in Human Immortalized Schwann and MPNST Cell Lines

**Figure 1:**
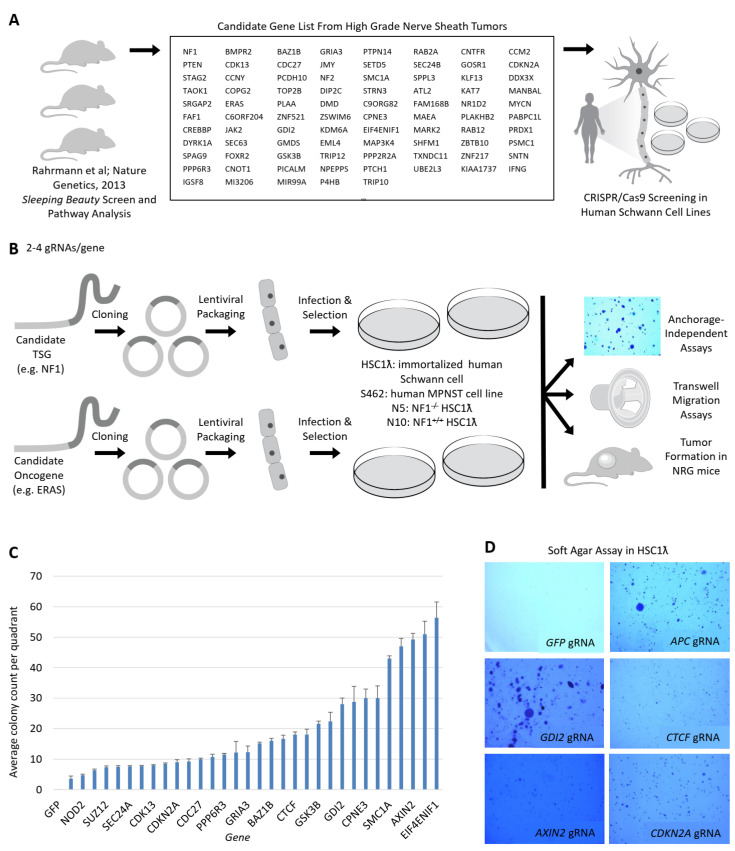
A streamlined approach to assess tumor suppressor gene candidates and oncogene function in human MPNST cell lines reveals novel MPNST pathways. (**A**) Diagram depicting summary of candidate selection from sleeping beauty (SB) screen to CRISPR/Cas9 forward genetic screening. (**B**) Diagram depicting screening method. First, gRNAs were designed to target genes selected in (**A**) and then stable cell lines were created and assessed for transformation upon genetic knockout. (**C**,**D**) show a summary of scores and anchorage-independent growth for predicted and known tumor suppressor gene candidates (1.5× magnification).

*SB* mutagenesis in Schwann lineage cells in mice that do not form genetically engineered mouse-PNSTs (GEM-PNSTs) identified over 100 candidate genes associated with aggressive GEM-PNSTs suppressor genes [11]. To understand the relevance of specific candidate genes in human Schwann cell transformation, we performed a medium-throughput screen (Figure 1A). The list of genes tested is shown in Figure 1A and Table S1. We used CRISPR/Cas9 technology to evaluate loss-of-function of candidate oncogenes and tumor suppressor genes in human immortalized Schwann and MPNST cell lines. We designed two to three guide RNAs (gRNAs) per candidate gene and cloned these gRNA sequences (Table S1) into an all-in-one lentiviral vector [12]. We included a gRNA against GFP as a negative control. Viral pools were generated that consisted of the gRNAs for each gene used to transduce cells, which were then selected in puromycin. Each generated cell population contained two to three gRNAs targeting a single candidate gene. They were expected to be a mixture of wildtype and knockout cells. Cell lines transduced included HSC1λ, TERT, and a murine CDK4-immortalized human Schwann cell line [3,11]. HSC1λ *NF1*^−^/^−^ were made using CRISPR/Cas9 [13] and S462 [14], an MPNST cell line derived from a lung metastasis of an NF1 patient. 

We first evaluated the effects of these pooled gene knockout cells on anchorage-independent growth in soft agar. We cultured cells for 14 days and then fixed, stained, and counted colonies per quadrant in duplicate (Figure 1B). *CDKN2A*, *NF1*, and *SUZ12* are known MPNST tumor suppressor genes [11] that served as positive controls in our screen. We found that 60% of TSG and 30% of oncogene candidates from the *SB* CIS (*SB* common insertion site)-associated genes scored in the soft agar screen (Figure 1C,D, Table S2). Several of these showed an increased transformative effect when knocked out in a *NF1*^−/−^ background, suggesting that they cooperate with the loss of *NF1*. These included *APC*, *TAOK1*, and *CCM2*. We also identified candidate oncogenes that reduced anchorage independent growth when knocked out in the MPNST cell line S462, which harbors a high mutational burden. These included *ERAS*, *ZNF521*, and *SRGAP2* (Figure S3). We focused on TSGs known to impact common signaling pathways. 

### 2.2. Tumor Suppressor Gene Candidates Suggest Multiple Pathways and Control Systems That Are Operative in the Schwann Cell Lineage

We performed both an ingenuity pathway analysis (QIAGEN Inc. Germantown, MD, USA,) [15] combined with a TSG data review that scored in the screen (Figure S1). Represented pathways included Wnt, Chromatin Dynamics, Ras, and cell cycle regulation pathways, as well as Hippo/Yes Activated Protein (YAP) and RhoA signaling. These genes included *NF2*, *TAOK1*, and *GDI2*. Wu et al. showed that *Lats1* and *Lats2* loss in mice results in Schwann cell tumor formation [16]. LATS1/2 phosphorylate YAP resulted in YAP cytoplasmic sequestration (Figure S1). All three genes downregulated at the mRNA level in a subset of MPNSTs compared to normal human Schwann cells. We identified downregulation in the expression of *TAOK1* in human MPNSTs). On the other hand, *GDI2*, a negative regulator of Rho signaling, is hypermethylated at its promoter, as found in MPNST human tumor samples [11]. This results in the downregulation of *GDI2* expression, which is predicted to lead to Rho activation [17]. Many genes scored in pathways active in the molecular mechanisms of cancer, as described by IPA pathway analysis (Figure S1). Other high-ranking pathways included Shh and DNA damage repair. 

### 2.3. Knockout of TAOK1 Results in Hippo/YAP Pathway Activation and Transformation

**Figure 2:**
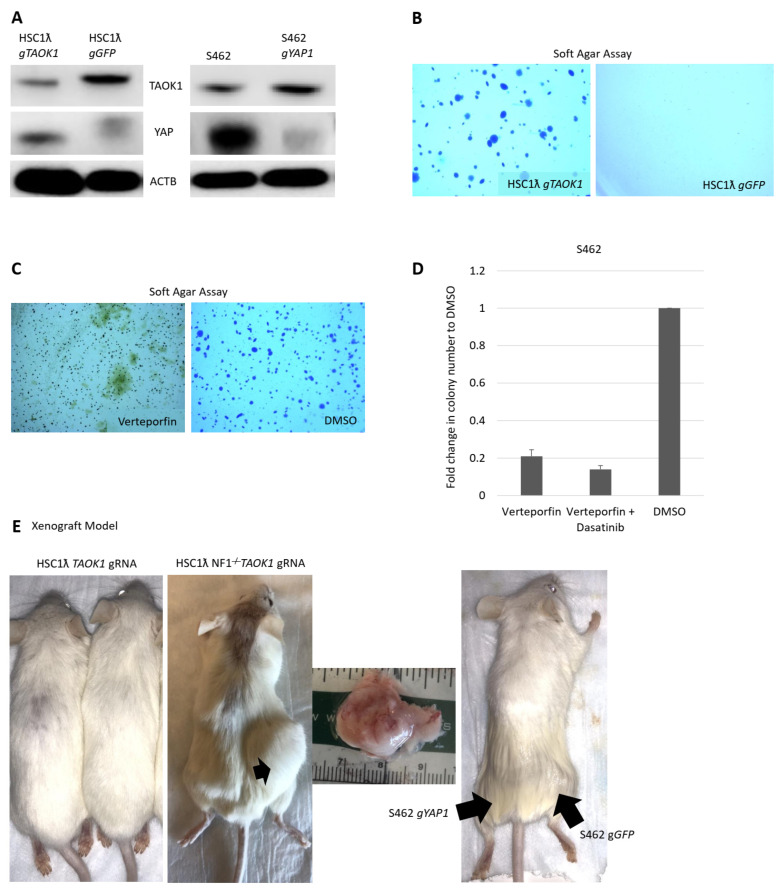
TAOK1 is a Schwann cell tumor suppressor gene. (**A**) Western blot showing TAOK1 decreased levels and increased YAP levels upon gRNA against *TAOK1* in a human immortalized Schwann cell line and loss of YAP Western blot in the MPNST cell line S462. (**B**) Anchorage-independent assay showing cell transformation upon *TAOK1* knockout in (**C**) and anoikis upon treatment with verteporfin (1.5× magnification). (**D**) Inhibition of YAP/TAZ activity and tyrosine kinase inhibition results in loss of anchorage-independent growth. (**E**) Tumor formation in NRG mice.

Wu et al. recently found that the loss of *Lats1/2* results in TAZ/YAP activation and tumor formation in Schwann lineage cells in mice [16]. However, pathways controlling YAP activation in human MPNSTs have not yet been described. We previously identified *Taok1* as a *SB*-CIS gene in the context of loss of *Nf1* (Figure 1A). *TAOK1* encodes a serine/threonine kinase that acts as a tumor suppressor via direct phosphorylation of YAP, preventing nuclear YAP translocation and instead resulting in cytoplasmic accumulation and degradation [11]. About 8% of MPNSTs have lost one or both copies of *TAOK1* [17]. The loss of *TAOK1* in immortalized human Schwann cells results in increased nuclear YAP1 levels (Figure 2A). Concomitant loss of *NF1* and *TAOK1* resulted in tumor formation in vivo (Figure 2C). To determine if YAP1 plays a role in MPNST cell survival, we knocked out this gene in S462 cells. The loss of *YAP1* in S462 cells decreased colony formation in soft agar and tumor formation in the S462 xenograft model (Figure 2B,C,E). To test if YAP is required in MPNSTs, we used the small molecule verteporfin, an inhibitor of YAP/TAZ binding [9]. Verteporfin reduced colony formation in soft agar (Figure 2D), suggesting a direct role for YAP signaling in tumor formation.

### 2.4. Loss of GDI2 Results in Rho Pathway Activation via Fak and Hippo/Yap Activation

**Figure 3:**
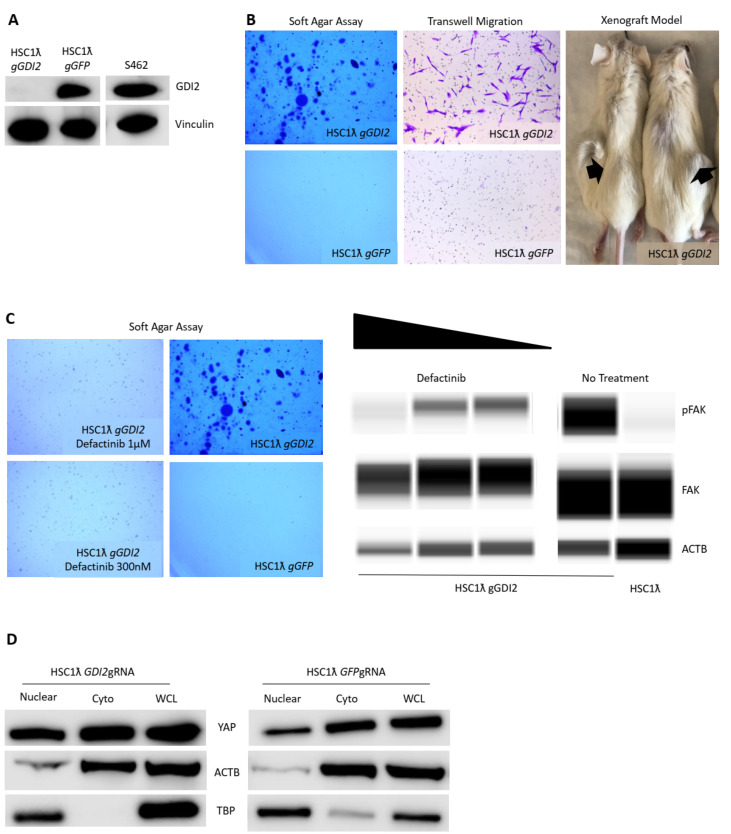
Rho is a MPNST pathway and its negative regulator GDI2 is a novel Schwann cell tumor suppressor gene. (**A**) Western blot showing knockout of *GDI2* in an immortalized Schwann cell line and levels in S462 MPNST cells. (**B**) Loss of *GDI2* results in transformation as seen by increased anchorage-independent growth cell migration and tumor formation in NRGs (1.5× magnification). (**C**) Loss of *GDI2* results in FAK activation and treatment with defactinib, a Rho inhibitor results in decreased anchorage-independent growth (2× magnification). Digital Western blot (Wess by Protein Simple) shows decreased FAK phosphorylation in GDI2-deficient cells upon treatment with defactinib. (**D**) Loss of *GDI2* results in increased YAP levels and nuclear localization.

The Rho signaling pathway is known to regulate cell cycle and polarity, adhesion, motility, and survival. *GDI2* is a member of the family of GDP-dissociation inhibitors. Its main function is to control the access of Rho GTPases to guanine exchange factors (GEFs) and GTPase activating proteins (GAPs) [18]. This, in turn, results in Rho activation and F-actin remodeling that alters cell adhesion and motility, as well as YAP nuclear translocation in what is thought to be a LATS-independent manner [19,20]. *GDI2* expression was downregulated in a subset of human MPNSTs via promoter hypermethylation and copy number loss [11]. The loss of *GDI2* (Figure 3A) led to increased migration in a transwell assay and tumor formation in a xenograft model (Figure 3B). *GDI2* was amongst the most robust tumor suppressor gene candidates to score in anchorage-independent growth (Figure 3B). To discover which Rho pathway effectors were active upon loss of *GDI2*, we used Western blotting to identify that FAK and ROCK were both active (phosphorylated) upon *GDI2* loss (Figure 3C). Drug studies revealed that inhibition of FAK or ROCK decreased the viability of MPNST cells and decreased anchorage-independent growth in soft agar (Figure 3C). Consistent with the role of actin dynamics in control of Hippo/Yap signaling, we observed that the loss of *GDI2* resulted in increased YAP protein levels in whole cell lysates and in a nuclear fraction (Figure 3D). 

### 2.5. Loss of APC Results in Wnt and Hippo/Yap Pathway Activation and Tumor Formation

**Figure 4:**
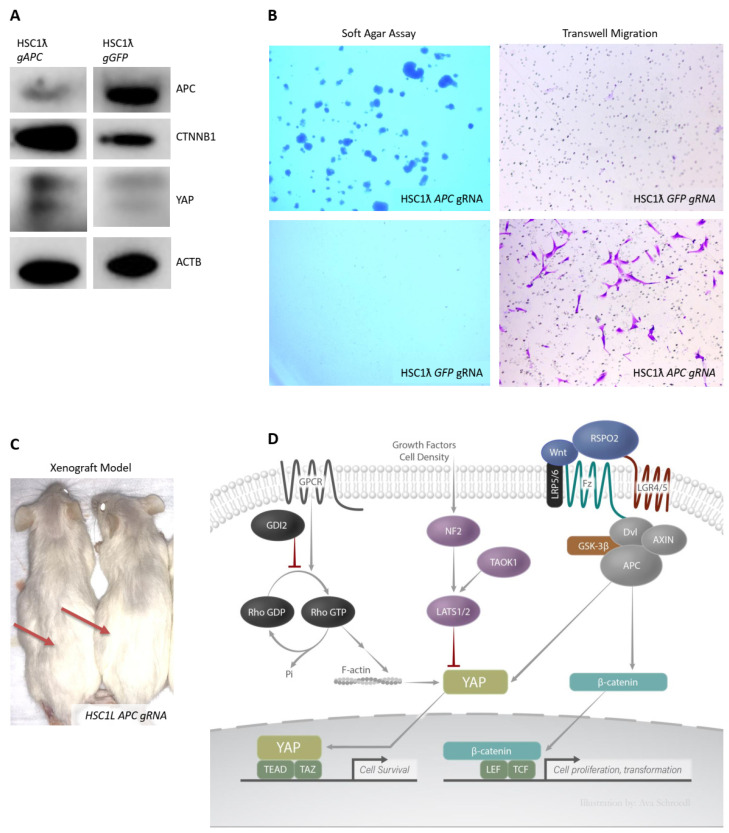
Wnt pathway negative regulator APC is a Schwann cell tumor suppressor gene and multiple pathways converge downstream resulting in Hippo/YAP activation. (**A**) Western blot showing loss of *APC* via CRISPR/Cas9 and increased YAP1 and CTNNB1 levels. (**B**) Loss of *APC* results in anchorage-independent growth and cell migration (1.5× magnification). (**C**) Loss of *APC* results is tumor formation in NRG xenograft model. (**D**) Diagram summarizing results showing overall Hippo/YAP activation via the loss of multiple tumor suppressor genes that occur in MPNSTs. Wnt, growth factor activity, cell density and Rho pathway(s) activation results in Hippo/YAP pathway activation in MPNSTs.

We and others previously described a role for the canonical Wnt pathway in MPNSTs [3]. We found that in vitro β-catenin activation results in increased Schwann cell proliferation and survival. In this study, we found that reduced expression of β-catenin destruction complex components such as *APC* and *GSK3B* is a common feature of MPNSTs [3]. The knockout of *APC* in immortalized Schwann cells (Figure 4A) resulted in increased colony formation in soft agar and increased migration in an anchorage-independent assay (Figure 4B). Moreover, we found that the loss of *APC* also resulted in tumor formation in a xenograft model (Figure 4C). It has been shown that Wnt/β-catenin signaling can activate the Hippo/Yap pathway as the APC-dependent destruction complex also regulates Yap stability [2]. Consistent with this prior report, we found that loss of *APC* in human Schwann cells resulted in increased levels of both CTNNB1 and YAP1 (Figure 4A).

## 3. Discussion

MPNSTs are genetically and epigenetically complex tumors. Several new agents are in clinical trials now, including MEK inhibitors, in some cases they are combined with a mTORC1/2 inhibitor or a BET/Bromodomain inhibitor [21]. Thus far, there have been no non-surgical strategies to induce a complete remission. Therefore, it urgent to discover new targetable MPNST tumor genes and pathways. In this study, we were able to validate the *SB*-CIS genes from our previous work in mice [11] using CRISPR/Cas9 knockouts in an immortalized human Schwann cell line. We observed the transforming effects of the knockout of known MPNST TSGs, including *CDKN2A*, *PTEN*, and *SUZ12*, confirming our methods. Our screen also implicated newly identified information, including Rho signaling [22]. 

Many of the TSG pathways we validated converge on YAP protein stabilization and nuclear translocation. Our results are consistent with the idea that the dysregulation of the Hippo/Yap pathway can occur via three different mechanisms in human Schwann cell tumors and MPNSTs: the loss of expression of Hippo negative regulators, such as *NF2* or *TAOK1*, Wnt signaling, and Rho modulation. The loss of *TAOK1* and/or *NF2* expression are frequent events in MPNSTs [11]. Wu et al. described the loss of expression of the negative Hippo/Yap regulators *LATS1* and *LATS2* in some human MPNSTs, as well as the knockout of *Lats1* and *Lats2* as potentially transforming for the Schwann lineage in mice [23]. Decreased expression may occur by gene copy number loss and/or promoter methylation. Indeed, we note that *TAOK1* is linked to *NF1*, located roughly 1000 Mb away. *SUZ12* is also genetically linked to *NF1*, and its loss is a known feature of MPNST [17]. In a subset of cases, loss of *TAOK1* occurs concomitantly with the loss of *NF1* and/or *SUZ12* [24]. This results in further YAP phosphorylation and its cytoplasmic sequestration (Figure 2A). *NF2*, another tumor suppressor gene, is known to negatively regulate the Hippo pathway [24], and we found that many MPNSTs have reduced *NF2* expression. 

Our work reinforces the notion that the activation of the Wnt pathway might contribute to MPNST development. Our results may also suggest that the APC destruction complex regulates stability of YAP, as well as β-catenin (Figure 4A). *APC* encodes the main negative regulator of Wnt signaling by acting as a scaffold for the β-catenin destruction complex [24]. *APC* was found to be lost in a subset (25%) of MPNSTs in the most recent TCGA Research Consortium analysis on soft tissue sarcomas [6]. YAP can also be bound by APC and phosphorylated for destruction by GSK3B [25]. Therefore, the loss of *APC* can result in the release of both β-catenin and YAP and their subsequent translocation to the nucleus. We hypothesize that the combined effects of β-catenin and Yap activation result in increased migration, inhibition of anoikis (i.e., anchorage independent growth), cell proliferation, and survival of Schwann tumor cells. 

The Rho pathway is also reported to regulate Hippo/Yap signaling [26]. The loss of Rho negative regulators occurs in a variety of human cancers, including MPNSTs [22]. Its context-dependent effects are not understood in MPNSTs. Hippo is known to be regulated in a mechanosensory fashion [26,27]. F-actin remodels downstream of Rho activation; in particular, it can result in changes of cell adhesion and shape with alteration of YAP nuclear levels in a LATS-independent manner. Therefore, the loss of *GDI2* may result in increased YAP nuclear levels via increased Rho activity. In fact, the loss of *GDI2* results in Rho activation via FAK and Hippo activation, as well as via increased YAP1 stabilization (Figure 3C,D). 

An examination of publicly available (TCGA) human RNA-seq data, CNA, and methylation revealed that *NF2* and *TAOK1* are deleted and under-expressed, the *GDI2* promoter is hypermethylated, and *APC* is lost in a subset of human cancers (Figure S2). Independently or together, activation of these upstream pathways by loss of TSG activity results in subsequent YAP activation (Figure 4D). These may be early or late events in the genesis and/or progression of plexiform neurofibromas to “atypical” neurofibroma and to MPSNT. 

## 4. Methods

### 4.1. Tissue Culture Reagents and Cell Lines

HSC1λ and S462s were a gift given to Dr. Margaret Wallace [28]. N5 and N10 cells lines underwent CRISPR/Cas9 targeting against *NF1*. The N5 cell line was derived from a clone found to have a deletion, resulting in the loss-of-function of *NF1*. The N10 cell line was derived from a clone in which *NF1* remained intact. Cells were cultured in DMEM supplemented with 10% FBS and 1% penicillin/streptomycin. All cells were grown on tissue culture-treated plates at 37 °C and 5% CO_2_. For in vitro drug studies, verteporfin and defactinib were solubilized to the desired concentration in DMSO independently.

### 4.2. CRISPR/Cas9 Knockout of Candidate Tumor Suppressor Genes and Oncogenes 

CRISPR/Cas9 modified cell lines were generated using lentiviral vectors expressing Cas9 and a guide RNA directed against each candidate gene. Lentiviral vectors were generated by transfecting 293T cells with two viral packaging plasmids and CC9 v2 Cas9/guide RNA-containing plasmid from the Zhang lab at MIT, Cambridge, MA, USA (lentiCRISPR v2 was acquired via Addgene plasmid # 5296). Guide RNA sequences were cloned into a stuffer region of the plasmid using Bsmb1 restriction sites. Guide RNA sequences to the gRNA can be found in Table S1. Sequences were designed to target downstream of the translational start site. 

### 4.3. Oncogenic Potential In Vitro and In Vivo Studies

Soft-agar assays were performed using a 0.48% low melting point agarose in sterile water. In total, 7500 cells were plated per well in a 6-well plate. Pictures were taken in dissecting microscope at 1.5× and analyzed using ImageJ. Xenograft models were performed in NRG mice and 3 million cells were injected subcutaneously in media containing Matrigel (1:1). Tumors were harvested, measured, and analyzed 4 months post-injection.

### 4.4. Western Blot Analysis 

A total of 2 million cells were lysed using a RIPA buffer, supplemented with a cocktail phosphatase inhibitor and a cocktail protease inhibitor. For nuclear fractions, a hypotonic solution supplemented with NP-40 was used to lyse the cells. Nuclear fractions were isolated via centrifugation and then lysed with a complete RIPA buffer. Whole-cell lysates and cell fractions were sonicated and prepared in solutions composed of SDS and a reducing agent, then boiled prior to size separation in either 10% or a gradient (4–12%) polyacrylamide gel (Invitrogen, Waltham, MA, USA). Gels were transferred to a PVDF membrane overnight. Membranes were then blocked in 5% non-fat milk TBST for 1 h at room temperature. Primary antibodies were diluted to manufacturers recommendation in 5% TBST milk or 5% BSA milk and incubated overnight at 4 °C. A list of antibodies and concentrations is available in Table S3. After being washed in TBST, HRP-conjugated secondary antibodies were then added and incubated at room temperature for 45 min, then developed using HRP chemiluminescence and visualized in a LICOR machine. 

## 5. Conclusions

Further analysis is necessary in humans at different stages of both benign and malignant Schwann cell tumors to determine at what stages these genetic events and pathways are regulated, and when they normally occur. MPNSTs harbor a variety of CNAs, some of which are recurrent and not yet successfully targeted for therapy. Given that the loss of *NF1* activates RAS-MEK, PI3K-mTOR, and other Ras-GTP-dependent signaling pathways, they make strong candidates for therapeutic targeting. Our research strongly suggests that multiple genetic events and aberrant pathway activation result in increased YAP1 activity, resulting in increased cancer cell survival via Hippo/YAP. We believe that other MPNST alterations, affecting other pathways, suggest that combination therapies should be attempted. Verteporfin has been used as a model small molecule to study YAP activity. Its use in humans is limited, but our studies show an increased need for development of a targeted Hippo/Yap inhibitor. In the absence of a viable YAP inhibitor, MPNSTs might be targeted with MEK inhibitor in combination with YAP, Rho, and/or Wnt inhibitors. Knowing the specific landscape of individual MPNSTs and defining common molecular subsets will shed light into the right combination of targeted therapies to use in a subset of patients. 

## Data Availability

The data presented in this study are available on request from the corresponding author.

## Supplementary Materials

The following are available online at https://www.mdpi.com/article/10.3390/cancers13071584/s1. Figure S1: IPA pathway analysis networks; Figure S2: Graph depicting average colony count in soft agar assay in the MPNST cell line S462; Figure S3: (A) Shows percentile scores of individual genetic knockouts in different human Schwann cell lines and the MPNST cell line S462. N10 cell lines is a clone derived from HSC1λ and N5 a HSC1λ clone that is *NF1* deficient. See Table S2 for further detail. (B) Table depicting scores of cell migration of CRISPR/Cas9 cells in a transwell assay; Table S1: gRNA sequences; Table S2: Results raw data; Table S3: Antibody list.
